# The NLRP3 Genetic Variant (rs10754555) Reduces the Risk of Adverse Outcome in Middle-Aged Patients with Chronic Coronary Syndrome

**DOI:** 10.1155/2022/2366695

**Published:** 2022-12-20

**Authors:** Trine B. Opstad, Jostein Nordeng, Alf-Aage R. Pettersen, Sissel Åkra, Vibeke Bratseth, Hani Zaidi, Ragnhild Helseth, Svein Solheim, Ingebjørg Seljeflot

**Affiliations:** ^1^Center for Clinical Heart Research, Department of Cardiology, Oslo University Hospital Ullevål, Oslo, Norway; ^2^Faculty of Medicine, University of Oslo, Norway; ^3^Department of Medicine, Vestre Viken HF, Ringerike Hospital, Hønefoss, Norway

## Abstract

**Background:**

Inflammation is central in development of cardiovascular disease (CVD). Aberrant function of the Nod-Like Receptor Protein 3 (NLRP3) inflammasome, a central mediator in the proinflammatory response, has been associated with atherosclerosis. The influence of genetic determinants on this inflammatory pathway and its downstream effects is less known. We aimed to investigate the frequency of a single NLRP3 gene variant according to clinical outcome in CVD and its influence on NLRP3-related markers.

**Methods:**

In this observational study, we included 1001 patients with chronic coronary syndrome. Blood samples were drawn at inclusion, including whole-blood and PAXgene tubes for DNA and RNA isolation, respectively. Allelic discrimination of the NLRP3 single nucleotide polymorphism rs10754555 was performed; and gene expression of NLRP3, Toll-Like Receptor 4, Interleukin- (IL-) 1*β*, and IL-18 was relatively quantified, both methods by RT-PCR. Circulating IL-6, high-sensitivity (hs) C-reactive protein, IL-18, and IL-12 were measured by enzyme-like immunosorbent assays. Clinical endpoints during 2 years (*n* = 106) were a composite of unstable angina pectoris, myocardial infarction, nonhemorrhagic stroke, and death.

**Results:**

Minor allele frequency of the NLRP3 variant was 0.36. In all, no association of the NLRP3 variant with clinical subgroups or outcome was found, neither any significant influence on the genes' mRNA expression or circulating protein. However, in subjects < 56 years (25 percentile), the variant G-allele is associated with significant lower risk of suffering a composite event (OR = 0.43 (95% CI 0.19, 0.97), *p* = 0.043, adjusted). In the same age group, the NLRP3 gene was accordingly downregulated in G-allele carriers vs. noncarriers, and circulating IL12 was significantly reduced (*p* < 0.05, both). In subjects > 56 years, no significant effect of the variant was observed.

**Conclusion:**

The age-related reduced risk of composite endpoint in rs10754555 G-allele carriers accompanied by diminished NLRP3 mRNA expression is hypothesis generating and needs to be further explored. The study is registered at http://www.clinicaltrials.gov, with identification number NCT00222261.

## 1. Introduction

Inflammation plays a pivotal role in the initiation and progression of atherosclerotic plaque and in the development of coronary artery disease (CAD). Several inflammatory signaling systems initiate the proinflammatory process, whereof the NOD-like receptor protein 3 (NLRP3) inflammasome/interleukin- (IL-) 1*β* pathway seems to be of importance. The NLRP3 inflammasome regulates the release of the proinflammatory cytokines IL-1*β* and IL-18, leading to the inception of a widespread inflammatory cascade, including the production of IL-6 and other acute phase reactants [1]. Inhibition of this signaling pathway has recently been shown beneficial in patients with CAD. In the CANTOS trial, the IL-1*β* antagonist canakinumab reduced cardiovascular risk in patients with established CAD, and the ASSAIL-MI study showed beneficial effect on myocardial salvation by the administration of tocilizumab, an IL-6R inhibitor, in patients with ST-elevation myocardial infarction (STEMI) [2, 3]. IL-18 in synergy with IL-12 is critical in the initiation and progression of Th-1-type responses by stimulating production of interferon-*γ* (IFN-*γ*), which induces multiple proatherogenic processes in the atherosclerotic lesion [4].

Aberrant activation and dysregulation of the NLRP3 inflammasome have been associated with several inflammatory disorders, including atherosclerosis [5]. It can be activated by pattern-recognition receptors (PRRs), including Toll-Like Receptor 4 (TLR4), which are stimulated by invading pathogens, dead cells, or environmental irritants, called either PAMPs (pathogen-associated molecular patterns) or DAMPs (damage-associated molecular patterns, including reactive oxygen species, triglycerides, cholesterol crystals, and cigarette smoking) [6, 7]. Upon activation, the PRRs initiate an upregulation of all NLRP3 inflammasome components via the nuclear translocation of nuclear factor- (NF-) *κ*B. After transcription, the NLRP3 assembles to a multiprotein complex, consisting of the NLRP3 protein, apoptosis-associated speck-like protein containing a caspase-recruitment domain (ASC), and pro-caspase-1. Once recruited into the complex, caspase-1 is activated and cleaves pro-IL-1*β* and pro-IL-18 into their active forms [8]. The regulation of NLRP3 inflammasome activity is not completely known, although mechanisms related to TLR4-mediated activation [9], posttranslational modifications [10], and NLRP3 interacting proteins have been described [11]. Transcriptionally, a NLRP3 genetic variant (rs10754555) affecting its NLRP3 mRNA levels in peripheral blood mononuclear cells (PBMCs) was recently reported to associate with NLRP3 inflammasome activation and increased systemic inflammation, CAD, and mortality in subjects below 60 years, the latter particularly in individuals with increased plasma levels of triglycerides [12].

The aim of the present study was to explore the frequency of the aforementioned NLRP3 genetic variant in a cohort of patients with chronic coronary syndrome (CCS) according to (1) clinical status and outcome after 2 years; (2) mRNA expression in circulating leukocytes of NL3P3, TLR4, and the downstream effector cytokines IL-1*β* and IL-18; and (3) circulating levels of IL-6, high-sensitivity (hs) C-reactive protein (CRP), IL-18, and IL-12. Any influence of age, triglycerides, and smoking status on the gene variants' impact on the mentioned outcomes was further assessed.

## 2. Material and Methods

### 2.1. Study Population

The study is a post hoc analysis of 1001 angiographically verified CCS patients enrolled in the ASpirin nonresponsiveness and Clopidogrel Endpoint Trial (ASCET), at Oslo University Hospital, Ullevål, Oslo, Norway, from 2004–2008 [13]. All patients were on aspirin at least one week prior to inclusion. Mean age was 62 years, 22% were females, and 97% were of Western European Descent. Patients were followed for a minimum of 2 years, and the primary clinical endpoint included the first event of a composite of nonfatal acute myocardial infarction (AMI), unstable angina pectoris (UAP), stroke, and all-cause mortality. No patients were lost to follow-up, and if unable to attend the final visit, clinical endpoints were recorded on request. An endpoint committee evaluated the endpoints without access to laboratory data.

At inclusion, clinical subgroups were defined as follows: previous MI as recorded by patients medical files, hypertension (HT) as previously diagnosed or treated HT, diabetes as individuals with treated diabetes type 2 (T2DM) and/or fasting glucose > 7.0 mmol/L, metabolic syndrome (Mets) according to modified NCEP criteria [14], and smoking habits as current smokers or not, with ex-smokers included as nonsmokers if they had quit smoking ≥3 months ago. The study was approved by the Regional Committee of Medical Research Ethics South-Eastern Norway. The participants conformed to the Declaration of Helsinki, and written informed consents were obtained from all. The ASCET study is registered at http://www.clinicaltrials.gov, with identification number NCT00222261.

### 2.2. Laboratory Sampling and Methods

Blood samples were drawn at inclusion by venipuncture between 08 : 00 and 10 : 30 a.m., in fasting condition and prior to morning medication. EDTA whole blood and PAXgene tubes were collected and frozen at -80°C for further DNA and RNA extraction, respectively. DNA was isolated using the MagNA Pure LC DNA isolation kit with the MagNA Pure LC Instrument (Roche Diagnostics, GmbH, Mannheim, Germany). Total RNA was isolated using the PAXgene Blood RNA kit (Qiagen GmbH for PreAnalytix, Hilden, Germany), with an extra cleaning step (RNeasy MinElute Cleanup kit, Qiagen). Both isolation procedures were performed according to the manufacturers' instruction. DNA and RNA purity and quantity were tested on the NanoDrop, ND-1000 (Saveen Werner, Sweden), and the nucleic acids were frozen at -80°C, until further analyses.

Serum was prepared by centrifugation within one hour at 2500 × g for 10 minutes and kept frozen at -80°C until analyzed. Blood samples for routine analysis (fasting glucose, HbA1c, and lipid profile) were analyzed using conventional methods. In serum, circulating IL-6, hs CRP, IL-18, and IL-12 were measured by Quantikine HS Human IL-6 (R&D Systems, Abingdon, UK), CRP HS ELISA (DRG Instruments GmbH, Germany), Human IL-18 ELISA (Medical Biological Laboratories, Naka-ku Nagoya, Japan), and Human IL-12 p40/p70 ELISA (Invitrogen, Thermo Fisher Scientific, Austria, Vienna). The interassay coefficients of variation for IL-6, hs CRP, IL-18, and IL-12 were 8.1%, 4.1%, 5.2%, and 5.8%, respectively.

#### 2.2.1. Genotype Analysis

Allelic discrimination of the NLRP3 variant (rs10754555, C ⟶ G substitution) was performed with real-time PCR on the ViiA™7 instrument (Applied Biosystems by Life Technologies, CA 92008 USA), using the TaqMan single nucleotide polymorphism (SNP) assay ID C_31451929_10 (Life Technologies dba Invitrogen, Pleasanton, California, USA) and TaqMan ™ Genotyping Master Mix (Applied Biosystems by Thermo Fisher Scientific, Baltics UAB, Lithuania). Nontemplate samples were included in each run to exclude contamination of samples, and 1% of the samples were reran as quality control with 100% concordance.

#### 2.2.2. Gene Expression Analyses

Gene expression of the NLRP3-related markers was performed in a subpopulation consisting of the first included 421 patients. Equal amounts of isolated total RNA per experiment (100 ng) were reversely transcribed into complementary (c) DNA by use of qScript cDNA SuperMix (Quanta Biosciences, Inc., Gaithersburg, USA). Gene expression was determined with PCR on the ViiA™ 7 Real Time PCR System, using TaqMan® Fast Universal PCR Master Mix (2X) No AmpErase® UNG, and the TaqMan® Gene Expression Assays for NLRP3 (Hs00918082_m1), TLR4 (Hs00152939_m1), IL-1*β* (Hs01555410_m1), and IL-18 mRNA (Hs00155517_m1) (Applied Biosystems, Foster City, CA, USA). The ∆∆Ct method was applied to determine the mRNA levels, using the *β*-2-microglobulin (HS99999907_m1) (Applied Biosystems) as the normalizer internal gene [15], validated as house-keeping gene in this population, and expressed as relative quantification (RQ) to a reference sample.

### 2.3. Statistical Methods

Data are presented as mean (±standard deviation (SD)), median (25th and 75th percentile), or proportions, as appropriate. Unpaired Student *t*-test and Mann–Whitney *U* test were used to compare continuous data with a normal or skewed distribution, respectively. Kruskal-Wallis test was used to compare skewed data across quartile groups. Proportional data were compared using Chi-squared test. In the age-stratified association between the NLRP3 gene variant and composite endpoint, a logistic regression model was performed, adjusting for sex by convention, and previous MI, differently distributed between those with and without an adverse outcome and due to its relevance for new clinical events. As no patient that suffered a stroke was ≤56 years at inclusion, and due to the limited number of subjects with an adverse outcome at the age ≤ 56 years (*n* = 26), previous stroke and other covariates were not included in the model. The allele and genotype frequencies of the NLRP3 rs10754555 gene variant were in Hardy Weinberg Equilibrium (HWE) (*x*^2^ > 0.05). *p* values < 0.05 were considered statistically significant. SPSS version 26 (SPSS Inc., IL, USA) was used for all statistical analyses.

## 3. Results

In this cohort of CCS patients, 106 clinical endpoints were recorded during 2 years; AMI (*n* = 36), stroke (*n* = 28), UAP (*n* = 33), and deaths (*n* = 9). Baseline characteristics according to presence or absence of clinical endpoints are presented in Table 1, showing previous MI and previous stroke to be more frequent in patients suffering a new clinical event. Presence of comorbidities in the total population was as follows: 20% with type 2 diabetes, 24% with Mets, 56% with HT, and 20% were current smokers. Medication status is shown in Table 1, equally distributed according to endpoints. Ninety-eight % of the patients were on statin treatment, with lipid values within the reference range.

### 3.1. Frequency of the NLRP3 Polymorphism (rs10754555) as Related to Future Adverse Events and Clinical Status at Baseline

DNA was available from 996 subjects, and the investigated NLRP3 rs10754555 gene variant was successfully analyzed in all. Distribution of the genotypes in the total population is presented in Table 2, showing that 41.6% were homozygous wild type (CC), 45.0% were heterozygous (CG), and 13.4% were homozygous of the variant allele (GG). The minor allele frequency (MAF) (G) was 0.36, in line with the report by Schunk et al. [12]. In the total population, the gene variant was not associated with new adverse clinical events, neither composite nor in subgroups, and no difference in G-allele frequency was detected between men and women (*p* = 0.68) (Table 2).  Table 3 presents the association of the rs10754555 variant with clinical status at time of inclusion. The G-allele was not associated with T2DM, Mets, previous MI, and stroke (*p* > 0.1, in all), although close to significant more frequent in overweight subjects (BMI > 27 kg/m^2^), *p* = 0.05.

**Figure 1:**
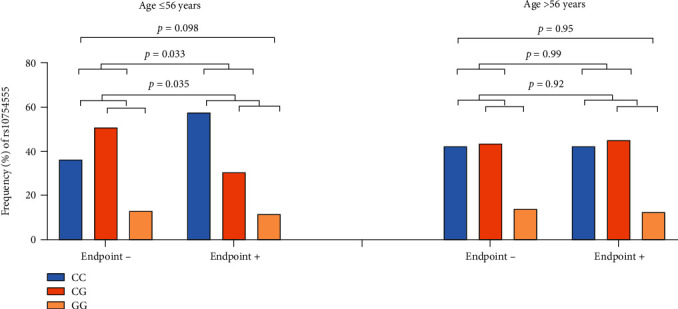
Frequency (%) of the NLRP3 (rs107545555 C/G) polymorphism according to clinical outcome dichotomized according to age at the 25 percentile level (56 years). Blue, orange, and pale orange columns represent homozygous wild type (CC), heterozygous (CG), and homozygous of the gene variant (GG), respectively. *p* values represent difference in genotype frequencies according to endpoint (chi square test) between CC, CG, and GG genotypes (2 × 3 table) between CC and CG genotypes (2 × 2 table), and between the CC genotype and the G-allele (CG/GG combined) (2 × 2 table).

Based on age differences regarding cardiovascular mortality in the publication by Schunk et al. [12], the risk of new clinical events as a function of NLRP3 rs10754555 variant was investigated according to age, dichotomized at the 25th percentile level (56 years) in our population.  Table 4 presents the difference in baseline characteristics between the actual age groups. At the age ≤ 56 years, the risk of suffering an adverse composite event differed significantly between the homozygous wild-type (CC) and heterozygous (CG) (*p* = 0.033). Also, in G-allele carriers (CG/GG) vs. noncarriers (CC), a significant lower risk of suffering an adverse composite event (OR = 0.42 (95% CI 0.18, 0.96), *p* = 0.035) was found (Figure 1). The association remained statistically significant after adjusting for sex and previous MI (OR = 0.43 (95% CI 0.19, 0.97), *p* = 0.043). At the age > 56 years, no significant association was observed between the genotypes and outcome (*p* > 0.9 in all) (Figure 1). As triglycerides and cigarette smoking can trigger the activation of the NLRP3 inflammasome, the G-alleles' impact on endpoint risk was investigated according to elevated triglycerides and smoking status. Lipid values did not differ between G-allele carriers and noncarriers (Supplementary Table (available here)). In subjects ≤ 56 years with triglyceride levels above median (1.31 mmol/L) (*n* = 142), the observed reduced risk was strengthened (OR = 0.27 (95% CI 0.09, 0.85), *p* = 0.025, adjusted). Likewise, in current smokers being ≤56 years (*n* = 80), the G-allele carriers were even stronger protected from suffering a composite endpoint (OR = 0.15 (95% CI 0.03 0.68), *p* = 0.014 adjusted).

### 3.2. Impact of the NLRP3 rs10754555 Variant on mRNA Expression and Circulating Levels of NLRP3 Inflammasome-Related Markers

**Figure 2:**
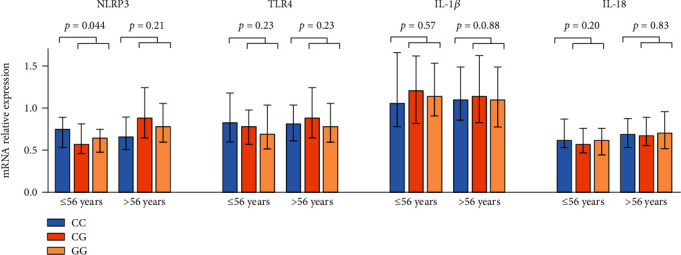
Change in gene expression (mRNA levels) of NLRP3, TLR4, IL-1*β*, and IL-18 according to NLRP3 genotypes. Blue, orange, and pale orange columns represent homozygous wild type (CC), heterozygous (CG), and homozygous of the gene variant (GG), respectively. Results are dichotomized according to age at the 25 percentile level (56 years). *p* values represent difference in gene expression of each marker between the CC genotype and the G-allele (CG/GG combined) (Mann–Whitney test).

mRNA expression in circulating leukocytes of the investigated markers (NLRP3, TLR4, IL-1*β*, and IL-18) according to the NLRP3 gene variant is shown in Table 5, comprising data from 421 subjects. We found no significant differences in expression of markers according to the NLRP3 AA, AG, and GG genotypes. However, in subjects ≤ 56 years, significantly lower NLRP3 mRNA expression in G-allele carriers compared to noncarriers was found (*p* = 0.044) (Figure 2). The other genes were not differently expressed accordingly.

**Figure 3:**
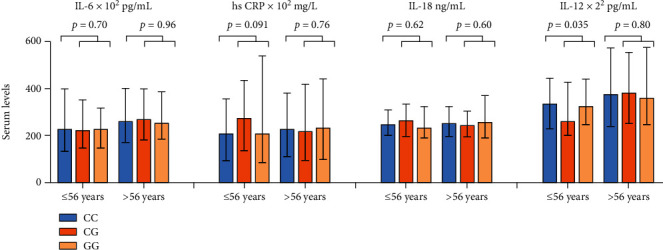
Levels of circulating IL-6, hs CRP, IL-18, and IL-12 according to NLRP3 genotypes Blue, orange, and pale orange columns represent homozygous wild type (CC), heterozygous (CG), and homozygous of the gene variant (GG), respectively. Results are dichotomized according to age at the 25 percentile level (56 years). *p* values represent difference in circulating levels of each marker between the CC genotype and CG/GG combined (Mann–Whitney test).

Circulating levels of IL-6, hs CRP, IL-18, and IL-12 were analyzed in 664, 994, 993, and 993 samples, respectively. Their concentration according to the NLRP3 AA, AG, and GG genotypes is presented in Table 6, showing no significant modifications. In subjects ≤ 56 years, significantly lower IL-12 levels were observed in G-allele carriers compared to noncarriers (*p* = 0.035) (Figure 3). The other measured markers were not modified accordingly (Figure 3).

## 4. Discussion

The main and novel finding in the present study was a reduced risk of adverse clinical events in CCS patients ≤ 56 years being carriers of the NLRP3 gene variant (rs10754555). In this age-group, NLRP3 mRNA expression was significantly downregulated in G-allele carriers vs. noncarriers. No such modification was observed at the age ≥ 56 years, and the variant did not influence mRNA levels of the other NLRP3 inflammasome-related components TLR4, IL-1*β*, and IL-18. Circulating levels of the inflammatory markers IL-6, hs CRP, IL-18, and IL-12 were not substantially influenced by the NLRP3 gene variant, except for significantly lower IL-12 concentration in G-allele carriers vs. noncarriers at the age ≤ 56 years.

The observed reduced risk of composite endpoint in association with the investigated NLRP3 gene variant has not previously been reported. The protective effect was only found at the age ≤ 56 years, indicating that more present cardiovascular risk factors at older age might overrule the beneficial effects of the polymorphism, i.e., the observed significant higher frequency of HT. NLRP3 mRNA expression and NLRP3 inflammasome activation are thought to decline with age [16]; thus, a reduced leukocyte NLRP3 expression in G-allele carriers might be more protruding at younger stages. Additionally, the higher medicinal use and frequency of women at the age > 56 years might have influenced the results, although only diuretics were more frequently used at this age, and the gene variant was not differently distributed between sexes. At the age ≤ 56 years, triglyceride levels were significantly higher compared to in older individuals, and despite being a trigger of NLRP3 activation, the risk of adverse events was significantly reduced in G-allele carriers with triglyceride levels above median (1.31 mmol/L). Chronic nutrient excess has been associated with elevated DAMP levels, and the synthesis of diacylglycerides to generate triglycerides from fatty acids is typical damage-associated metabolites that activate the NLRP3 inflammasome [17]. The observed G-alleles' protective effect might be explained by such chronic exposure.

At the age ≤ 56 years, significantly more subjects were current smokers compared to older ages, and a numerically higher number of smokers was found in G-allele carriers vs. noncarriers. Despite a suggested NLRP3 inflammasome activation with cigarette smoking [7], the polymorphism significantly prevented adverse events in current smokers. Cigarette smoking has on the contrary been reported to inhibit the NLRP3 inflammasome, accompanied by macrophage dysfunction [18]. If so, the G-allele might have reinforced this effect. Altogether, the observed results exemplify gene-environment interactions and indicate that lifestyle inducers of the NLRP3 inflammasome may impact the association between the NLRP3 gene variant and clinical outcome.

Our finding is supported by the significantly reduced NLRP3 mRNA expression and IL-12 concentration in G-allele carriers ≤ 56 years compared to noncarriers at similar age. Although no effect of the G-allele was observed on TLR4, IL-1*β*, and IL-18 mRNA expression and IL-6, hs CRP, and IL-18 concentrations, the downregulated NLRP3 protein might have influenced the activity of the NLRP3 inflammasome, with less systemic inflammation and subsequently lower risk of adverse events.

Our findings contrast the observed increased risk of all-cause and cardiovascular mortality in subjects with this polymorphism as shown in the LURIC study, including 3061 participants at the age < 60 years undergoing coronary angiography, particularly in presence of elevated triglycerides [12]. With a three times higher number of investigated subjects than in our study, the diverging result is difficult to explain. Moreover, the initial findings in the LURIC study were validated in 10 prospective clinical trials in subjects with or without preexisting CAD, with 6% and 14% increase in risk of cardiovascular mortality, respectively, as a function of the rs10754555 polymorphism. Moreover, the polymorphism was associated with increased NLRP3 mRNA expression in PBMCs in the LURIC study, also validated in 36 additional cohorts [12]. Our conflicting results are not easily explainable. The different populations might not be comparable, and the previous results combined multiple studies in a meta-analysis. In the initial study by Schunk et al., results were not adjusted for classical CVD risk factors. Our results with regard to clinical events were adjusted for potential confounders such as previous MI, and despite the limited number of subjects in our study, the results were significant. The performed allelic discrimination analysis was validated by repeated measurements, with 100% concordance. Our genotype analysis conformed to HWE, an important quality control step in population genetic studies, which most likely can exclude genotyping error [19]. Regarding the gene expression analyses, our measurements in circulating leukocytes were performed unstimulated in contrary to the stimulated experiments with isolated PBMC in the referred article by Schunk et al. [12], which might have contributed to the contradictory results. The reported elevated plasma IL-18 and IL-1*β* levels and IL-1*β* release in the supernatant of PBMCs in G-allele carriers were also performed after stimulation, the latter in mice experiments transfected with human PBMCs [12]. That being said, hs CRP and serum amyloid A were observed elevated as a function of the NLRP3 rs10754555 variant in participants of the LURIC study [12].

Another modifier of the present results might have been medication status, as our population was heavily medicated, and, e.g., aspirin is known to downregulate the NLRP3 inflammasome [20], whereas statin may both stimulate and inhibit the inflammasome, partly dose-dependent [21]. However, the use of medication did not differ in subjects with or without a composite endpoint nor in the actual age subgroups except for diuretics.

### 4.1. Limitations

Several limitations need to be considered in our study. Although statistically significant results were achieved, the sample size was limited, in total and in subgroups, and the results should be interpreted with caution. Gene expression data were only performed in 42% of the included subjects, which may have led to selection bias. The inclusion of only one single polymorphism is a restraint, and the potential influence of other genetic variants in linkage disequilibrium with the rs10754555 variant can also not be excluded. As our population was homogenous, with 97% of Western European Descent, the results cannot be generalized to other ethnicities.

## 5. Conclusion

The G-allele of the investigated NLRP3 rs10754555 variant associated with reduced risk of composite endpoint in subjects with CCS at the age below 56 years, accompanied by diminished NLRP3 mRNA expression and IL-12 concentration. The results, achieved age-stratified, are hypothesis generating and need to be further explored.

## Figures and Tables

**Table 1:** Baseline characteristics in the population (*n* = 996) according to occurrence of composite clinical endpoint during two years.

Baseline characteristics	CV endpoints (*n* = 106)	No CV endpoints (*n* = 890)	*p*
Age (years, mean (range))	62 (41-80)	63 (36-81)	0.51
Men/women (*n* (%))	83/23 (78/22)	695/195 (78/22)	0.960
Type 2 diabetes mellitus (*n* (%))	24 (23)	175 (20)	0.468
Previous MI (*n* (%))	57 (54)	378 (42)	**0.027**
Previous stroke (*n* (%))	6 (6)	20 (2)	**0.037**
Hypertension (*n* (%))	63 (59)	493 (55)	0.428
Current smokers (*n* (%))	23 (22)	178 (20)	0.646
BMI (kg/m^2^)^∗^	26.8 (24.4, 29,9)	27.2 (24.9, 29.6)	0.676
Metabolic syndrome	25 (24)	219 (25)	0.812
Total cholesterol (mmol/L)	4.5 (1.0)	4.6 (1.0)	0.870
HDL cholesterol (mmol/L)	1.3 (0.4)	1.3 (0.4)	0.898
LDL cholesterol (mmol/L)	2.5 (0.8)	2.5 (0.8)	0.749
Triglycerides (mmol/L)^∗^	1.25 (1.02, 1.87)	1.32 (0.93, 1.84)	0.869
Fasting glucose (mmol/L)	6.1 (1.7)	6.0 (1.9)	0.931
Medication (%)			
Statins	99	98	0.520
*β*-Blockers	74	76	0.868
Nitrates	27	21	0.154
ACE inhibitors	30	26	0.324
ARB	25	24	0.735
CCB	27	26	0.680
Diuretics	26	23	0.435
Insulin	6	4	0.510
Oral antidiabetics	11	12	0.859
No antidiabetics	8	6	0.454

Values are mean (SD) or number (proportions) if not otherwise stated. MI: myocardial infarction; SD: standard deviation; BMI: body mass index; HDL: high-density lipoprotein; LDL: low-density lipoprotein; ACE: angiotensin converting enzyme; ARB: angiotensin receptor blocker; CCB: calcium channel blocker. *p* values are chi-square test for categorical variables and *t*-test and Mann–Whitney test for continuous variables, referring to differences between patients with and without endpoints. Gem median levels (25 and 75 percentiles). Bolded texts indicate statistical significance.

**Table 2:** Frequency of the NLRP3 variant in the total population and according to type of new clinical events and sex.

		CC	CG	GG	G-allele frequency	*p* value
Total cohort^∗^		414	448	134	0.360	
With clinical event	+	49	44	13	0.331	0.303
	-	365	404	121	0.363	
UAP	+	19	9	5	0.289	0.058
	-	395	439	129	0.362	
AMI	+	19	14	3	0.278	0.164
	-	395	434	131	0.362	
Stroke	+	8	17	3	0.411	0.157
	-	406	431	131	0.358	
Death	+	3	4	2	0.444	0.615
	-	411	444	132	0.359	
Men		326	344	108	0.360	0.684
Women		88	104	26	0.358	

^∗^Number of available DNA samples = 996. UAP: unstable angina pectoris; AMI: acute myocardial infarction. *p* values represent the difference in G-allele frequency (CG and GG combined) according to subgroups (chi-square test).

**Table 3:** Frequency of the NLRP3 variant according to comorbidity at baseline.

		CC	CG	GG	G-allele freq.	*p* value
Previous MI	+	192	186	57	0.345	0.147
	-	222	262	77	0.375	
Previous stroke	+	11	13	2	0.327	0.942
	-	403	434	132	0.360	
Diabetes	+	78	89	32	0.385	0.448
	-	336	359	102	0.353	
Mets	+	101	110	33	0.360	0.938
	-	313	337	101	0.359	
Hypertension	+	228	246	82	0.368	0.687
	-	186	202	52	0.348	
Current smoker	+	75	102	24	0.373	0.177
	-	338	346	110	0.359	
BMI ≤ 27 kg/m^2^		222	214	61	0.339	0.050
BMI > 27 kg/m^2^		192	233	73	0.381	

MI: myocardial infarction; Mets: metabolic syndrome; BMI: body mass index dichotomized at the median level. *p* values represent the difference in G-allele frequency (CG and GG combined) according to presence of comorbidity (chi-square test).

**Table 4:** Baseline characteristics of the total population according to age dichotomized at 56 years.

Baseline characteristics	≤56 years (*n* = 248)	>56 years (*n* = 752)	*p* value
Men/women (*n* (%))	210/38 (85/15)	572/180 (76/24)	**0.004**
Type 2 diabetes mellitus (*n* (%))	46 (19)	154 (20)	0.51
Previous MI (*n* (%))	112 (45)	325 (43)	0.59
Previous stroke (*n* (%))	0 (0)	27 (3.6)	**0.002**
Hypertension (*n* (%))	110 (44)	445 (59)	**<0.001**
Current smokers (*n* (%))	80 (32)	123 (16)	**<0.001**
BMI (kg/m^2^)^∗^	27.4 (24.8, 30.1)	27.0 (24.8, 29.4)	0.23
Metabolic syndrome	62 (25)	182 (24)	0.81
Total cholesterol (mmol/L)	4.6 (1.1)	4.5 (0.9)	0.25
HDL cholesterol (mmol/L)	1.2 (0.4)	1.4 (0.4)	**<0.001**
LDL cholesterol (mmol/L)	2.6 (0.9)	2.5 (0.8)	0.27
Triglycerides (mmol/L)^∗^	1.47 (1.03, 2.24)	1.26 (0.91, 1.76)	**<0.001**
Fasting glucose (mmol/L)	6.1 (2.3)	6.0 (1.8)	0.75
Medication (%)			
Statins	243 (98)	739 (98)	0.91
*β*-Blockers	177 (71)	580 (77)	0.20
Nitrates	46 (19)	171 (23)	0.17
ACE inhibitors	61 (25)	202 (27)	0.50
ARB	8 (3)	36 (5)	0.09
CCB	60 (24)	195 (26)	0.62
Diuretics	35 (14)	190 (25)	**<0.001**
Insulin	10 (4)	34 (6)	0.75
Oral antidiabetics	26 (10)	92 (12)	0.46
No antidiabetics	14 (6)	46 (6)	0.79

MI: myocardial infarction; SD: standard deviation; BMI: body mass index; HDL: high-density lipoprotein; LDL: low-density lipoprotein; ACE: angiotensin converting enzyme; ARB: angiotensin receptor blocker; CCB: calcium channel blocker. *p* values are chi-square test for categorical variables and *t*-test and Mann–Whitney test for continuous variables, referring to differences between patients with and without endpoints. ^∗^ Median levels (25 and 75 percentiles). Bolded texts indicate statistically significant *p* values.

**Table 5:** Relatively quantified gene expression (mRNA levels) of NLRP3, TLR4, IL-1*β*, and IL-18 genes as related to NLRP3 genotypes in the total population.

	CC	CG	GG	*p* ^1^ value	*p* ^2^ value
NLRP3	0.666 (0.522, 0.894)	0.674 (0.470, 0.917)	0.709 (0.529, 0.950)	0.820	0.865
TLR4	0.815 (0.605, 1.093)	0.846 (0.610, 1.182)	0.746 (0.581, 1.055)	0.419	0.709
IL-1*β*	1.095, (0.829, 1.545)	1.168 (0.819, 1.614)	1.123 (0.855, 1.499)	0.777	0.668
IL-18	0.680 (0.530, 0.876)	0.660 (0.525, 0.870)	0.664 (0.520, 0.849)	0.884	0.620

*p* ^1^ values represent the difference between genotypes (Kruskal-Wallis test). *p*^2^ values represent the difference between homozygous wild type (CC) and the G-allele (CG and GG combined) (Mann–Whitney test).

**Table 6:** Circulating levels of markers as related to NLRP3 genotypes in the total population.

Protein in serum	CC (*n* = 414)	CG (*n* = 448)	GG (*n* = 134)	*p* value^1^	*p* value^2^
IL-6 pg/mL	2.53 (1.66, 3.96)	2.49 (1.70, 3.83)	2.51 (1.64, 3.70)	0.932	0.712
Hs CRP mg/L	2.23 (1.08, 3.71)	2.28 (1.03, 4.22)	2.17 (0.97, 4.60)	0.823	0.547
IL-18 ng/mL	251 (198, 320)	246 (195, 315)	245 (188, 342)	0.854	0.832
IL-12 pg/mL	93 (59, 133)	86 (58, 132)	86 (62, 131)	0.610	0.353

*p* ^1^ values represent the difference between genotypes (Kruskal-Wallis test). *p*^2^ values represent the difference between homozygous wild type (CC) and the G-allele (CG and GG combined) (Mann–Whitney test).

## Data Availability

Data can be available upon reasonable request to the main author.

## Supplementary Materials

Supplementary Materials Supplementary Table: serum lipid levels according to NLRP3 genotypes.

## References

[1] An N., Gao Y., Si Z. (2019). Regulatory mechanisms of the NLRP3 inflammasome, a novel immune-inflammatory marker in cardiovascular diseases. *Frontiers in Immunology*.

[2] Ridker P. M., Everett B. M., Thuren T. (2017). Antiinflammatory therapy with canakinumab for atherosclerotic disease. *The New England Journal of Medicine*.

[3] Broch K., Anstensrud A. K., Woxholt S. (2021). Randomized trial of interleukin-6 receptor inhibition in patients with acute ST-segment elevation myocardial infarction. *Journal of the American College of Cardiology*.

[4] Hansson G. K., Robertson A. K., Soderberg-Naucler C. (2006). Inflammation and atherosclerosis. *Annual Review of Pathology*.

[5] Kelley N., Jeltema D., Duan Y., He Y. (2019). The NLRP3 inflammasome: an overview of mechanisms of activation and regulation. *International Journal of Molecular Sciences*.

[6] Takeuchi O., Akira S. (2010). Pattern recognition receptors and inflammation. *Cell*.

[7] Mehta S., Srivastava N., Bhatia A., Dhawan V. (2020). Exposure of cigarette smoke condensate activates NLRP3 inflammasome *in vitro* and *in vivo*: a connotation of innate immunity and atherosclerosis. *International Immunopharmacology*.

[8] Fernandes-Alnemri T., Wu J., Yu J. W. (2007). The pyroptosome: a supramolecular assembly of ASC dimers mediating inflammatory cell death via caspase-1 activation. *Cell Death And Differentiation*.

[9] Yang Y., Lv J., Jiang S. (2016). The emerging role of toll-like receptor 4 in myocardial inflammation. *Cell Death & Disease*.

[10] Yang J., Liu Z., Xiao T. S. (2017). Post-translational regulation of inflammasomes. *Cellular & Molecular Immunology*.

[11] Piippo N., Korhonen E., Hytti M. (2018). Hsp90 inhibition as a means to inhibit activation of the NLRP3 inflammasome. *Scientific Reports*.

[12] Schunk S. J., Kleber M. E., März W. (2021). Genetically determined NLRP3 inflammasome activation associates with systemic inflammation and cardiovascular mortality. *European Heart Journal*.

[13] Pettersen A. A., Seljeflot I., Abdelnoor M., Arnesen H. (2012). High on-aspirin platelet reactivity and clinical outcome in patients with stable coronary artery disease: results from ASCET (aspirin nonresponsiveness and clopidogrel endpoint trial). *Journal of the American Heart Association*.

[14] Malik S., Wong N. D., Franklin S. S. (2004). Impact of the metabolic syndrome on mortality from coronary heart disease, cardiovascular disease, and all causes in United States adults. *Circulation*.

[15] Opstad T. B., Pettersen A. A., Weiss T. W. (2012). Genetic variation, gene-expression and circulating levels of matrix metalloproteinase-9 in patients with stable coronary artery disease. *Clinica Chimica Acta*.

[16] Connat J. L., Dumont A., Rialland M., Faivre B., Sorci G. (2018). Nlrp3 gene expression in circulating leukocytes declines during healthy aging. *The Journals Of Gerontology Series A, Biological Sciences And Medical Sciences*.

[17] Camell C., Goldberg E., Dixit V. D. (2015). Regulation of Nlrp3 inflammasome by dietary metabolites. *Seminars in Immunology*.

[18] Buscetta M., Di Vincenzo S., Miele M., Badami E., Pace E., Cipollina C. (2020). Cigarette smoke inhibits the NLRP3 inflammasome and leads to caspase-1 activation via the TLR4-TRIF-caspase-8 axis in human macrophages. *FASEB journal: official publication of the Federation of American Societies for Experimental Biology*.

[19] Yu C., Zhang S., Zhou C., Sile S. (2009). A likelihood ratio test of population Hardy-Weinberg equilibrium for case- control studies. *Genetic Epidemiology*.

[20] Zhou X., Wu Y., Ye L. (2019). Aspirin alleviates endothelial gap junction dysfunction through inhibition of NLRP3 inflammasome activation in LPS-induced vascular injury. *Acta Pharmaceutica Sinica B*.

[21] Koushki K., Shahbaz S. K., Mashayekhi K. (2021). Anti-inflammatory action of statins in cardiovascular disease: the role of inflammasome and toll-like receptor pathways. *Clinical Reviews in Allergy and Immunology*.

