# Supplementary material for: The NLRP3 Genetic Variant (rs10754555) Reduces the Risk of Adverse Outcome in Middle-Aged Patients with Chronic Coronary Syndrome
Source: J Immunol Res. 2022 Dec 20;2022:2366695. doi: 10.1155/2022/2366695 (PMC9794420; doi:10.1155/2022/2366695)
Supplement: Supplementary Materials — Supplementary Table: serum lipid levels according to NLRP3 genotypes. [file 2366695.f1.docx]

**Supplementary Material**

**Supplementary Table** Serum lipid levels according to NLRP3 genotypes

|  | CC | CG/GG | p-value |
| --- | --- | --- | --- |
| Total cholesterol mmol/L | 4.6 (1.0) | 4.5 (1.0) | 0.82 |
| HDL cholesterol mmol/L | 1.2 (0.4) | 1.3 (0.4) | 0.67 |
| LDL cholesterol mmol/L | 2.5 (0.8) | 2.5 (0.8) | 0.95 |
| Triglycerides mmol/L* | 1.30 (0.93, 1.88) | 1.32 (0.94 1.83) | 0.94 |

Values are mean (SD)

* Median values (25, 75 percentiles)
